# Schrödinger and the Possible Existence of Different Types of Life

**DOI:** 10.3389/fmicb.2022.902212

**Published:** 2022-05-31

**Authors:** Greco Hernández

**Affiliations:** From the Translation and Cancer Laboratory, Unit of Biomedical Research on Cancer, National Institute of Cancer (Instituto Nacional de Cancerología, INCan), Mexico City, Mexico

**Keywords:** What is life?, life definition, synthetic life, viral life, alien life, origin of life

## Abstract

Eighty years ago, Nobel Prize-winner physicist Erwin Schrödinger gave three lectures in Dublin’s Trinity College, titled *What is Life? The physical aspect of the living cell* to explain life in terms of the chemistry and physics laws. Life definitions rely on the cellular theory, which poses in the first place that life is made up of cells. The recent discovery of giant viruses, along with the development of synthetic cells at the beginning of century 21st, has challenged the current idea of what life is. Thus, rather than having arrived at a close answer to Schrödinger’s question, modern biology has touched down at a novel scenario in which several types of life—as opposed to only one—actually might exist on Earth and possibly the Universe. Eighty years after the Dublin lectures, the Schrödinger question could be: “What are lives”?

## What is Life?

In 1935, German physicist Max Delbrück wanted to study “the universal phenomenon that governs life,” i.e., genetic replication. He asked a fundamental question in biology: How can physics laws explain gene extraordinary stability throughout generations? (Timoféeff-Ressovsky et al., 1935; Delbrück, 1946). Influenced by Delbrück’s ideas, on February 5th, 1943, Austrian physicist Erwin Schrödinger gave a series of lectures at Trinity College, Dublin, titled *What is Life? The physical aspect of the living cell*, in which he also emphasized the thermodynamic aspect of life: The fact that genes and organisms are highly ordered structures, apparently remaining outside thermodynamic equilibrium, seems to defy the second law of thermodynamics, which poses that everything in the Universe is decaying into disorder (Olby, 1994). How do genes and living entities seem to escape the cosmic tide that sweeps everything into ultimate chaos? Schrödinger asked the question “what is life?”

Schrödinger’s lectures were published in a book titled *What is Life?* (Schrödinger, 1944), which became very influential in biology. Over the next decades, hundreds of definitions of life have been proposed basically relying on the cellular theory which poses that (1) living entities are made up of cells; (2) every cell is originated by another cell; and (3) cells are the basic unit of all living organisms. Interestingly, in the years 2000–2004, two major breakthroughs stirred biology, namely, the discovery of giant viruses by the French school of virologists (Forterre, 2010; Claverie and Abergel, 2016; Colson et al., 2017; Abergel and Claverie, 2020), and the *in vitro*-developed synthetic organisms by Craig Venter’s team in the United States of America (Venter, 2014). Ever since, these discoveries have challenged the concepts of what life is, putting forward the revolutionary idea that more types of life might exist on Earth. In addition, the search for life in the cosmos has intensified in the last few years. If discovered, alien life’s nature might be different from the one on our planet. Thus, it seems that life’s puzzle has multiplied. Eighty years after the lectures at Dublin, the Schrödinger question has become: “What are the lives”?

## Viruses and Life

Viruses are overwhelmingly the most abundant and diverse biological entities on the planet, and practically all living beings are infected by a broad spectrum of viruses (Suttle, 2007, 2013; Villarreal and Witzany, 2010; Koonin and Dolja, 2013; Paez-Espino et al., 2016; Gregory et al., 2019). Ever since viruses were discovered in 1898 (Beijerinck, 1898; Löffler and Frosch, 1898), the debate on whether they are alive or not was born. Because their biology so different from cells, researchers asked, “what is a virus”?

In 2003, French virologists discovered a weird virus infecting amoebae in a water tank of a hospital in Bradford, England, which was named mimivirus (La Scola et al., 2003; Raoult et al., 2004). Interestingly, its biology did not match that of known viruses (Colson et al., 2017), as mimivirus is 100 times larger than the viruses known back then and even larger than many bacteria. Moreover, its genome contains hundreds of genes, many of them key to cellular metabolism thought to be present “only” in cells (Colson et al., 2017). While common viruses contain between 4 and 15 genes, mimivirus has 1,018 and pithovirus (another giant virus) more than 2,550 genes, 90% of which did not resemble any known gene at their discovery (Colson et al., 2019). In 2008, La Scola and Raoult further discovered that giant viruses in turn, are infected by other viruses called “virophages,” something that was supposed to happen “only” to cells (La Scola et al., 2008; Colson et al., 2017). Back then, none of this made sense under the classical idea of what a “virus” is, i.e., particles 100–500 times smaller than bacteria, containing few genes, and never infected by other viruses (Raoult and Forterre, 2008; Colson et al., 2019).

Over time, new giant viruses and virophages have been discovered in many other environments infecting amoebae, microalgae, and dinoflagellate (Krupovic et al., 2016; Abergel and Claverie, 2020). When a mimivirus infects an amoeba, a well-defined compartment called the “virion factory” is formed in its cytoplasm, where the components of the new virions are synthesized and assembled. At this time, mimiviruses behave and resemble very much intracellular parasitic bacteria (which are living cells) (Claverie, 2006). Claverie and others proposed that, just as parasitic organisms need another species to complete their life cycle, viruses are also living organisms that require a host cell for the same purpose (Claverie, 2006; Raoult and Forterre, 2008; Claverie and Abergel, 2010, 2016; Forterre, 2010; Koonin and Dolja, 2013; Dupré and Guttinger, 2016).

The discovery of giant viruses and virophages led French virologists to strongly question the current meaning of “virus” and asked again, a century after having been discovered, “what is a virus?” They also made broader questions like: are viruses alive? Do we need to redefine the concept of “life”? (Claverie, 2006; Raoult and Forterre, 2008; Raoult, 2009; Claverie and Abergel, 2010, 2016; Forterre, 2010, 2016; Dupré and Guttinger, 2016; Colson et al., 2017, 2019).

No definition of “life” exists, but there is a consensus that “living things” have at least three characteristics: metabolism, genetic material, and reproductive capacity. However, “living thing” is a concept established by cell theory decades before viruses were discovered. Indeed, viruses collide with such definition, and mimiviruses force us to rethink what life is. Moreover, viruses have genes, proteins, replicate, express their genetic information, evolve by Darwinian processes to adapt to their habitats, interact with their environment, play critical roles in cell evolution, strongly influence Earth biogeochemistry, occupy ecological niches in all ecosystems, and were probably key for the origin and early evolution of life. Thus, Claverie proposed the radical notion that giant viruses are alive and that their life consists of a phase of “temporarily living organism” (the viral factory inside an infected cell), and an inert phase (the viral particle or virion) that serves as a vehicle to transport its genes from cell to cell (Claverie, 2006; Claverie and Abergel, 2016). Raoult and Forterre set forward the idea that viruses might conform a type of life different than the cell-based life and further proposed the existence of two kinds of life on Earth: a viral life and a cellular life (Raoult and Forterre, 2008).

## Life and Synthetic Biology

The American biochemist Paul Berg and colleagues developed in the 1970s the powerful technology to fuse DNA from different species, the so-called “recombinant DNA.” DNA manipulation gave rise to synthetic biology (SB), which applies engineering principles and design to living organisms to artificially create novel biological systems and communities (Baldwin et al., 2016; Kriegman et al., 2020). SB rationally engineers novel genotypes and phenotypes for specific technical applications utilizing a biological chassis, i.e., a host cell that is genetically modified by introducing modular functional DNA units. Nowadays, SB is a crucial research field with a ∼$12 Bn investment in the United States and United Kingdom since 2015 for industrial and biotechnological applications (Freemont, 2019; Clarke and Kitney, 2020). SB achievements are groundbreaking and of enormous impact on humankind. Ultimately, the design of living organisms at the DNA level has created parallel biodiversity.

In 2010 Craig Venter artificially created the first bacterium with a synthetic genome in history, *Mycoplasma genitalium* JCVI-1.0 (Gibson et al., 2010), by transplanting a complete genome manufactured by a machine to the natural *M. genitalium*. Recently, the same team created the bacterium *Mycoplasma mycoides* with an artificially reduced minimal genome consisting of 452 genes that can sustain growth and self-replication. This genome is smaller than any natural viable independent cell. This synthetic bacterium was dubbed “JCVI-syn3A” (Hutchison et al., 2016; Breuer et al., 2019).

In eukaryotes, scientists further substituted a chromosome of the budding yeast *Saccharomyces cerevisiae* with a highly modified, synthetic version (Richardson et al., 2017). Moreover, gene networks and metabolic pathways have been redesigned in many species (Baldwin et al., 2016; Meng and Ellis, 2020). The genetic code itself has also been *in vitro* engineered. It has been expanded or reduced in different organisms by stop codons suppression and codon reassignment to synthesize a vast catalog of proteins with unnatural amino acids (Wang, 2017; Fredens et al., 2019; Kato, 2019). Moreover, space SB is currently engineering various biological systems for space exploration and future human settlements on different extraterrestrial bodies. Soon, space SB will design artificial organisms able to live in extreme environments, i.e., bioreactors undergoing wide variations in temperature, ionizing, and minimal nutrient supplies (Menezes et al., 2015). However, we still do not know what type of organisms these will be.

With the development of SB, the conception of the living must now contemplate synthetic life, namely organisms possessing different regions of the genome (even the complete genome) or the genetic code designed by man.

## On the Possibility of Discovering Alien Life

Astrobiologists’ goal is to search for life in the cosmos. It is thought that life might appear on a body in the Universe once favorable physical-chemical conditions are established. However, the possible alien lives might have been created under exotic environments and thus could be based on chemistries utterly different from life on Earth (Bains, 2004; Benner et al., 2004; Schulze-Makuch and Irwin, 2008; Gagler et al., 2022). Hence, a formal definition of “life” is not possible for astrobiologists since we do not know what life will be like elsewhere in the cosmos and because we only have the sole example of Earth life (Cleland, 2019a,b; Gagler et al., 2022). Indeed, extraterrestrial life could blur even more the current Earth-centric definitions of life.

The current discovery of thousands of exoplanets has fueled the search for life in outer space. The development of technology to detect possible signs of alien life (i.e., “biosignatures”) has also accelerated using Earth-based telescopes and space-based technology carried on spatial missions. Biosignatures are defined as an object, substance, and/or pattern whose origin specifically requires a biological agent (Des Marais et al., 2008). However, without a definition of life based on different possible biochemistries beyond Earth, identifying alien life is a very challenging task. Furthermore, to understand what life is, we need to know the broad fundamental principles that rule life across the Universe. Nevertheless, life origins should be driven by probabilistic events led by thermodynamic constrictions (Lehman and Kauffman, 2021).

To date, accepted biosignatures include atmospheric oxygen (O_2_), ozone (O_3_), methane (CH_4_), nitrous oxide (N_2_O), and methyl chloride (CH_3_Cl), chemical disequilibrium within an atmosphere, low entropy, and non-random high abundance of specific complex compounds. Potential surface biosignatures are pigments for photo-protection and the temporal variation of these signatures (Hedge et al., 2015; Seager and Bains, 2015; Catling et al., 2018; Fujii et al., 2018; Kiang et al., 2018; Schwieterman et al., 2018; Walker et al., 2018).

Moreover, measuring molecular complexity as an indicator of life anywhere has been explored (Nikolić et al., 2003; Marshall et al., 2021), as living entities appear to be the sole systems able to produce highly complex molecules in abundance. Other features of any alien life are the requirement of energy to drive metabolic reactions, a liquid solvent to mediate such reactions, nutrients to increase biomass and system complexity, and the ability to produce catalytic molecules that drive metabolic reactions (Schwieterman et al., 2018).

Some of the methods to detect biosignatures include tandem mass spectroscopy for measuring paths of molecular assembly with high orders of magnitude of complexity (Marshall et al., 2021); measurements of Doppler-shifted lines due to the planetary atmosphere; spectroscopic signals; reflected light; changes of gas concentrations; metrics for chemical disequilibrium; assessment of spectral signatures of gases; photometric light curves analyses; thermal emission spectrometry; transmission spectroscopy; and direct-imaging using adaptive optics or coronagraphy (Hedge et al., 2015; Seager and Bains, 2015; Catling et al., 2018; Fujii et al., 2018; Kiang et al., 2018; Schwieterman et al., 2018; Walker et al., 2018).

## Origins of Life on Earth

How did life originated on Earth? Researchers investigate how the earliest chemical processes on Earth led to the emergence of the first living cells, which further evolved to become the last universal common ancestor (LUCA), and different models of prebiotic evolution have been set forward. Although a discussion on the origin of life is beyond the scope of this article, here I mention some of the most relevant research on this topic.

Genetic material-first scenarios focus on the origin of polymer replication and polymers that carry genetic information. In contrast, the metabolism-first supporters investigate what toolkit of chemical reactions were firstly able to sustain life. One of the most prolific scenarios is the proposal of an RNA World, as it answered the age-old chicken-and-egg question about whether DNA or proteins arose first in early cells.

Back in 1980, it was discovered that precursors of ribosomal RNA and the RNA moiety of ribonuclease P catalyze chemical reactions such as enzymes do. These catalytic RNAs were called “ribozymes” (Zaug and Cech, 1980; Cech et al., 1981; Guerrier-Takada et al., 1983). That is, RNA can act both as a carrier of genetic information and as a catalyst for chemical reactions. Thus, Walter Gilbert proposed that the first cells could have based their biology on catalytic RNA molecules that carried genetic information and, at the same time, catalyzed their own duplication. Gilbert named this hypothetical scenario “The RNA World” (Gilbert, 1986). The discovery of catalytic RNA made it very clear that life did not need a genetic code, proteins, or DNA to appear (Robertson and Joyce, 2012).

Alternative chemical models to the RNA World have been proposed. According to one of the most established, life could have arisen on mineral deposits in the porous rocks of the alkaline hydrothermal vents in the depth of the oceans. This microenvironment is rich in sulfur, iron, nickel, manganese, magnesium, and other mineral crucial for life. This model, named the “Iron-Sulfur World,” was proposed by Günter Wächtershäuser and further developed alongside Martin Russell and colleagues. In it, iron-sulfur minerals might have catalyzed the synthesis of organic molecules, and early life could have been sustained by the proton gradients emerging across the micropores’ mineral walls, which generate high amounts of energy. In this deep, dark, and mineral Garden of Eden, the existence of molecules that carry information was not necessary for the early cells to emerge (Wächtershäuser, 1990, 2007; Russell et al., 1998; Martin and Rusell, 2007; Martin et al., 2008; Huber et al., 2012).

Another interesting scenario is the proposal of sets of autocatalytic organic compounds that auto replicate as transitory intermediates between chemical systems and genetically encoded enzymes. Networks of autocatalytic molecules might have been the first-self-sustaining, autotrophic metabolic networks might have preceded proteins and RNA in the beginning of life (Kauffman, 1986; Smith and Morowitz, 2004; Hordijk and Steel, 2010; Xavier et al., 2020).

### Life in the Shadows?

Life on Earth emerged due to favorable physical-chemical conditions that prevailed on our early planet. Therefore, life might have arisen independently several times on Earth, and alternative extant forms of life are consistent with prevailing models of the origin of life (Cleland, 2019b). It has been recently proposed that our planet might host microbial descendants of, at least, a second life genesis, forming a sort of parallel, “shadow biosphere,” existing alongside the known biosphere, which is a descendant of a single, monophyletic LUCA (Cleland and Copley, 2005; Cleland, 2007, 2019b; Davies et al., 2009; Davies, 2011). If a second form of life existed in the past or still exists today, it might be different from the known biosphere.

The lack of evidence of life in the shadows might be due to the tools currently used to investigate the microbial world, namely microscopy, cultivation in specific growth media, and metagenomic methods which could be inadequate to explore cells of a different nature (Cleland, 2019b). However, it might be among us.

## On the Definition of Various Possible Types of Life

Next year, we will celebrate 80 years since Schrödinger asked *what is life*? Currently, rather than giving a compelling answer, modern biology has arrived at an unexpected scenario in which multiple types of life could exist on Earth and possibly the Universe. Thus, modern biology does not seem to have reached any closer to resolving Schrödinger’s enigma. Instead, we have moved away from it due to the highly complex panorama of what living organisms are.

Cell theory was established as a conceptual framework to understand the nature of living organisms before viruses were discovered and SB was born, not to say alien lives (if they are ever found). The current concepts of life and what it means to “be alive” are primarily based on cell theory and define terrestrial organisms, which are descendants of a common ancestor representing thus a single example of life (Cleland, 2019a). These definitions exclude viruses and certain organisms designed by SB, not to say the possible life in the shadows and perhaps extraterrestrial life forms.

Besides cellular, viral, and synthetic lives, it is impossible to know how many kinds of life might exist in the Universe and what their nature could be (Gagler et al., 2022). Does modern biology need to develop several definitions of life—as opposed to only one—for the Universe? In the future, do we need to answer the Schrödinger question?

## Author Contributions

GH conceptualized and wrote the whole manuscript.

## Conflict of Interest

The author declares that the research was conducted in the absence of any commercial or financial relationships that could be construed as a potential conflict of interest.

## Publisher’s Note

All claims expressed in this article are solely those of the authors and do not necessarily represent those of their affiliated organizations, or those of the publisher, the editors and the reviewers. Any product that may be evaluated in this article, or claim that may be made by its manufacturer, is not guaranteed or endorsed by the publisher.
